# Novel Approach of Nanostructured Bainitic Steels’ Production with Improved Toughness and Strength

**DOI:** 10.3390/ma13051220

**Published:** 2020-03-09

**Authors:** Peter Kirbiš, Ivan Anžel, Rebeka Rudolf, Mihael Brunčko

**Affiliations:** 1 Faculty of Mechanical Engineering, University of Maribor, Smetanova 17, SI-2000 Maribor, Slovenia; ivan.anzel@um.si (I.A.); rebeka.rudolf@um.si (R.R.); mihael.bruncko@um.si (M.B.); 2 SIJ Metal Ravne d.o.o., Koroška cesta 14, SI-2390 Ravne na Koroškem, Slovenia

**Keywords:** nanostructured bainitic steel, bainitic ferrite, microstructure, impact toughness

## Abstract

The tendencies of development within the field of engineering materials show a persistent trend towards the increase of strength and toughness. This pressure is particularly pronounced in the field of steels, since they compete with light alloys and composite materials in many applications. The improvement of steels’ mechanical properties is sought to be achieved with the formation of exceptionally fine microstructures ranging well into the nanoscale, which enable a substantial increase in strength without being detrimental to toughness. The preferred route by which such a structure can be produced is not by applying the external plastic deformation, but by controlling the phase transformation from austenite into ferrite at low temperatures. The formation of bainite in steels at temperatures lower than about 200 °C enables the obtainment of the bulk nanostructured materials purely by heat treatment. This offers the advantages of high productivity, as well as few constraints in regard to the shape and size of the workpiece when compared with other methods for the production of nanostructured metals. The development of novel bainitic steels was based on high Si or high Al alloys. These groups of steels distinguish a very fine microstructure, comprised predominantly of bainitic ferrite plates, and a small fraction of retained austenite, as well as carbides. The very fine structure, within which the thickness of individual bainitic ferrite plates can be as thin as 5 nm, is obtained purely by quenching and natural ageing, without the use of isothermal transformation, which is characteristic for most bainitic steels. By virtue of their fine structure and low retained austenite content, this group of steels can develop a very high hardness of up to 65 HRC, while retaining a considerable level of impact toughness. The mechanical properties were evaluated by hardness measurements, impact testing of notched and unnotched specimens, as well as compression and tensile tests. Additionally, the steels’ microstructures were characterised using light microscopy, field emission scanning electron microscopy (FESEM) and high-resolution transmission electron microscopy (HRTEM). The obtained results confirmed that the strong refinement of the microstructural elements in the steels results in a combination of extremely high strength and very good toughness.

## 1. Introduction

In order to meet the industries ever rising demands for tool and construction materials, new developments of advanced engineering steels are aimed at the simultaneous improvements of strength, ductility, and toughness. A traditional method commonly applied to meet such requirements is the refinement of the grain size, which is often quoted as the only means by which strength and toughness can be improved simultaneously, and has, thus, been at the core of material development within the past century. Its merits are apparent from such examples as commercially pure iron, whose strength increases from an initial 250 MPa to as high as 1500 MPa by refining the grain size from 20 µm to 0.25 µm by means of SPD (severe plastic deformation) [1]. A finer grain size also presents additional benefits by making the steel less susceptible to impurities [2], and enabling the refinement and accelerated formation kinetics of different phases [3]. The extent of strengthening achieved via grain refinements is described by the Hall-Petch equation relating grain size $D_{GB}$ to the yield strength $\sigma_{y}$:

(1) $$\sigma_{y}=\sigma_{0}+k_{1}/\sqrt{D_{GB}}$$

where $k_{1}$ is a material constant (usually close to 0,15 $MPa\sqrt{m}$ for steels) and $\sigma_{0}$ is the flow stress of an undeformed single crystal oriented for multiple slip, or approximately the yield stress of very coarse-grained, untextured polycrystalline metal.

The strength is expected to be directly proportional to the grain size, and while grain refinement is undoubtedly important, there are severe technological difficulties for the obtainment of grains approaching the nanoscale in bulk dimensions. Grain refinement is traditionally obtained through deformation, whereby a higher deformation ratio correlates roughly with a finer grain size. As metals soften with increasing temperature, they are deformed while red-hot, but as they tend to recover and recrystallize rapidly, the achievable refinement is limited due to the release of internal heat. Therefore, in order to obtain the desired nanostructures, it is necessary to apply deformation at low temperatures approaching the ambient level.

As materials deform additional strengthening is obtained, by increasing the amount of dislocations as described by the following relation [4]:

(2) $$\sigma_{\rho }=\frac{MGb\sqrt{\rho_{f}}}{2}$$

where $\rho_{f}$ is the density of free dislocation in the matrix, (dislocations whose movement isn’t restricted by other microstructural constituents), M is the Taylor factor (usually 3 for steels), and Gb are the shear modulus and Burgers vector respectively; for first approximation, values of 80 GPa and 0.25 nm may be used.

Further strengthening is then obtained due to the interaction of dislocations with various defects and atoms in a solid solution. Martensite and bainite formation introduces a high amount of interfaces, as well as mobile geometrically necessary dislocations, providing strengthening inversely proportional to the thickness of the transformation product. Upon tempering, the martensite laths structure transforms into a subgrain structure with free dislocations within the subgrains [5], and the strengthening contribution of elongated subgrains is proportional to the short distance [6]:

(3) $$\sigma_{sg}=\frac{10Gb}{\lambda_{sg}}\approx \frac{100}{\bar{L}}$$

where $\lambda_{sg}$ is the width of elongated subgrains, which corresponds to the width of martentic plates.

If we insert come typical values for steels into the above equation (10 Gb≈200), we can estimate the contribution of subgrain strengthening.

The subgrain model predics that the strengthening is proportional to ½ L, whereby L is the mean linear intercept between glide planes.

For bainitic steels which are not tempered, the Cottrell model is often thought to be more accurate, which evaluates the strengthening on the basis of expansion of dislocation loops, developed on the observations of deformation of fibrous structures in heavy drawn iron wires [7]:

(4) $$\sigma_{bl}=\frac{2.5Gb}{\bar{D}}\approx \frac{115}{\bar{L}}$$

where $\bar{L}$ denotes mean linear intercept (µm).

The true ferrite plate thicknesses *t* are determined by measuring the mean lineal intercept $\bar{L}=\;\frac{\pi t}{2}$ in a direction normal to the plate length.

The strengths of steels achievable with a variety of methods are summarised in Table 1, in correlation with their characteristic length and the volume fraction of internal boundaries.

When comparing the different strengthening mechanisms, there appears to be a seeming contradiction to the theory described earlier. Firstly, when the grains become extensively refined, there is an observed deviation from the Hall-Petch relations, whereby the grain size exponent changes from 0.5 to about 0.7 [19].

More importantly, one has to keep in mind certain saturation effects/limitations of the specific strengthening mechanism applied within a given alloy. For example, exceptionally fine grain sizes of about 100nm were obtained by (accumulative roll bonding) ARB processing of an (Interstitial Free) IF steel at room temperature. This procedure resulted in the development a strength level of 909 MPa [20], and, despite a substantial additional refinement of the order of four times, the value obtained is similar to the IF steel [16] quoted in Table 1. IF steel is the material of choice in many ARB experiments, due to its excellent plasticity and economic potential. However, there are little to no solute elements present to stabilise the grain boundaries, which would, thereby, make them more effective obstacles to dislocation movement. On the other hand, a high carbon pearlitic wire drawn to 20 nm grain size has been known to achieve exceptionally high strength, even up to 7 GPa, which is largely attributed to segregations and solid solution [21]. Parallel lines can also be drawn with other processes relying on severe plastic deformation, whereby it should be considered that ball milled steels are additionally strengthened by nanoscale oxide particles [22,23], which stabilise the grain boundaries and can introduce dislocation loops giving rise to strain hardening. 

It can be seen that the strength levels obtained via phase transformations of bainite [24] and martensite [25,26] are comparable, or even higher, compared to those commonly obtainable by SPD methods, whereby a significant portion of the work hardening capacity can potentially be retained, due to the presence of fine metastable retained austenite, and perhaps, more importantly, mobile dislocations. The presence of metastable austenite in itself is not sufficient to promote plasticity, as a severe drop in ductility with increasing deformation has also been observed in reverse transformed martensite in severely deformed austenitic steels, giving rise to very fine nanostructured grains [13].

Another practical advantage of great interest is that this approach, at the same time, enables the production of large cross-sections of complex shapes. There is also little indication that these mechanisms would be inherently limited, as these structures produce a glissille interface and a high amount of mobile dislocations. The nanostructured martensitic grades are appealing, but comparably little data are available, especially about the scaling of dimensions compared to their fine bainitic counterparts.

In this context, a novel group of bainitic steels is introduced, which exhibit a rapid development of carbide free lower bainite. Rapid formation of bainite has been observed at unprecedentedly low temperatures, even below 100 °C, whereas the transformation kinetics at room temperature are in the order of a few weeks. This is accomplished by a method the authors termed kinetic activation, refering to the process by which nuclei for bainite formation are formed at high temperature, enabling the rapid growth of bainitic ferrite at low temperatures, therefore these steels and their resulting microstructure, are referred to as kinetically activated bainite (KAB). 

This phenomenon is exciting, as, by virtue of the low transformation temperatures, the individual bainitic ferrite plates become exceptionally refined, ranging from a few tenths of nanometers to as little as a few nanometers. Common fields of aplication include rolls, bearings, and other machine parts subjected to rolling contact fatigue. Industrial knives and balistic protection. The goal of the current work is to examine the microstructural development in such steels in relation to heat treatment, and to evaluate the mechanical properties which are obtainable. Thereby, determining the optimal heat treatment route is achieved for the development of a of nanostructured bainitic microstructure within KAB steels.

## 2. Materials and Methods 

Experimental alloys were produced in the form of ingots (each 10 kg). Ingots were vacuum induction melted from commercially pure steels and alloying additions. In the current work, an alloy with the following nominal chemical composition in Table 2 is considered:

Ingots were forged to final dimensions of 80 mm × 6 mm. After forging, the billets were allowed to cool in air to room temperature, followed by annealing and final heat treatment by quenching in oil from 920 °C and tempering at 200 °C. Samples for testing of tensile properties and impact toughness were then EDM (electrical discharge machining) (SODICK AG 600 L Wire Eroding Machine, Schaumburg, IL, USA ) cut, and ground to final dimensions according to ASTM A370, and tested in compliance with the said Standard. Hardness values were measured using the Rockwell C method acc. to ASTM E 18-07. Samples for light microscopy were ground using SiC paper to P2400, followed by polishing using 3-µm and 1-µm diamond suspensions, the final step being 0,05µm alumina suspensions. For FESEM characterization (Sirion NC 400. Philips/FEI, Hillsboro, OR, USA ), the metallographically prepared samples were etched with Vilella gold, sputtered and observed using a Sirion NC 400.

Samples for high resolution transmission electron microscopy (HRTEM) (HRTEM is JEol JEM-2100 from JEol, Akishima Japan), observations were cut into 3-mm disks of 1-mm thickness on a slow speed saw and ground carefully to about 0.5 mm, followed by additional thinning using the procedure of electro polishing and light etching, and observed using a Jeol JEM-2100.

The phase fractions of ferrite and retained austenite were determined using X-ray diffraction on a Bruker D8 Advance (Bruker AXS Advanced X-ray Solutions GmbH, Karlsruhe, Germany), operating using a Cu anode at 40 kV/40 mA and a secondary graphite monochromator, within a 2 theta range between 40° and 90° for a total measuring time of 3 h. Prior to measurements, the samples were deep-etched in order to remove any deformed surface layer. 

**Figure 1:**
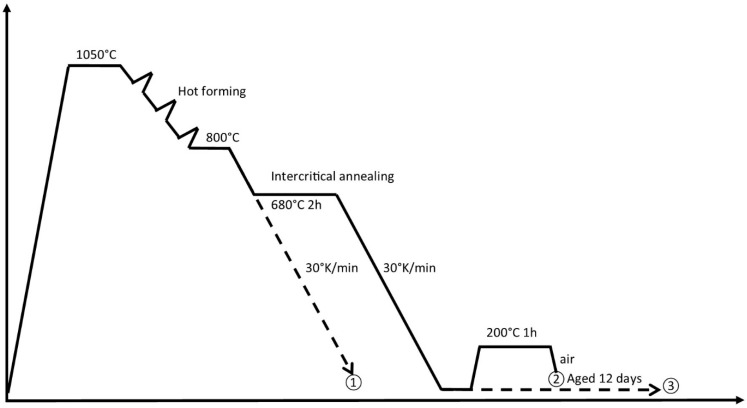
Different heat treatments and obtainable hardness levels of steel A0.

Three heat treatment routes following hot forming have been studied for steel A0, which are depicted in Figure 1:

Route ① is simple continuous cooling to room temperature, and serves as a reference point. Heat treatment route ② is aimed at increasing the stability of the retained austenite phase, which is known to be critical to the mechanical behaviour of mixed microstructures, whereas route ③ omits the retained austenite from the microstructure almost entirely, which is thought to give rise to other combinations of mechanical properties.

## 3. Results

### 3.1. Strength

The most apparent benefit of nanostructured materials is the increase in strength, and the levels of strength that can be obtained are directly correlated to the heat treatment.

**Figure 2:**
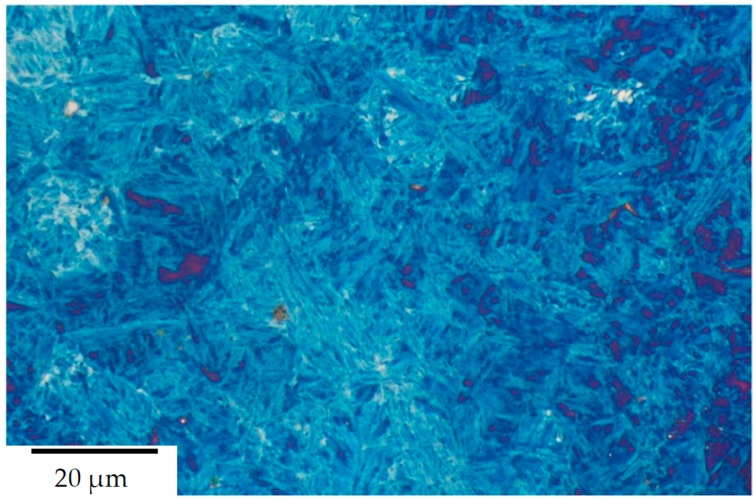
Light micrograph of steel A0 after heat treatment acc. to route ①. (Etched with 7% Na_2_S_2_O_5_, bainite is coloured blue, retained austenite dark/violet).

**Figure 3:**
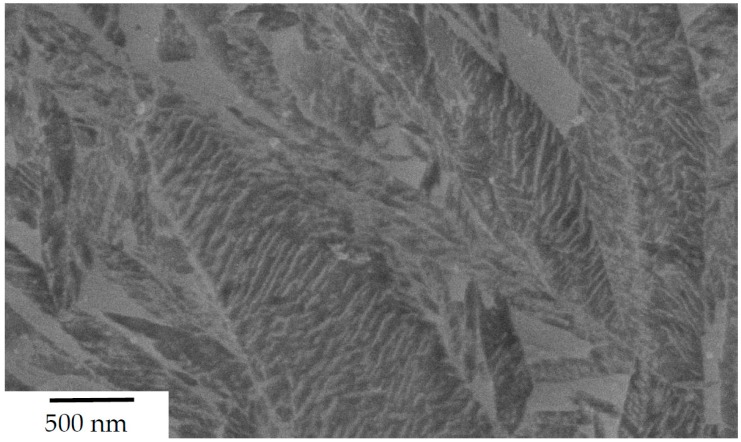
FESEM micrograph of steel A0 after heat treatment acc. to route ①.

**Figure 4:**
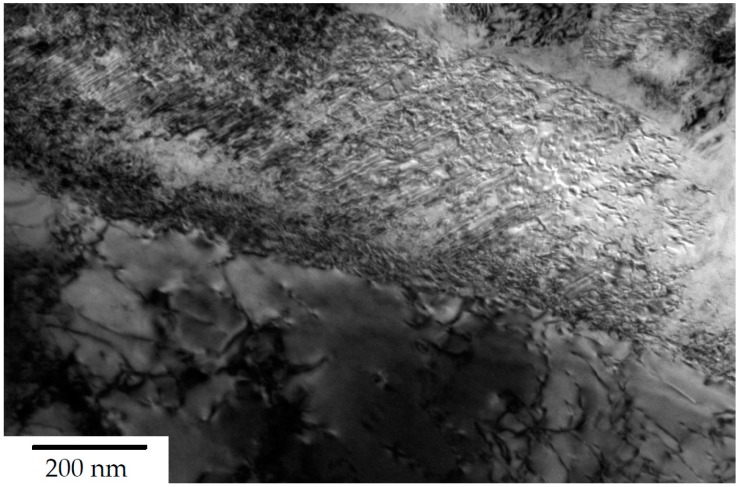
HRTEM micrograph of steel A0 after heat treatment acc. to route ①.

**Figure 5:**
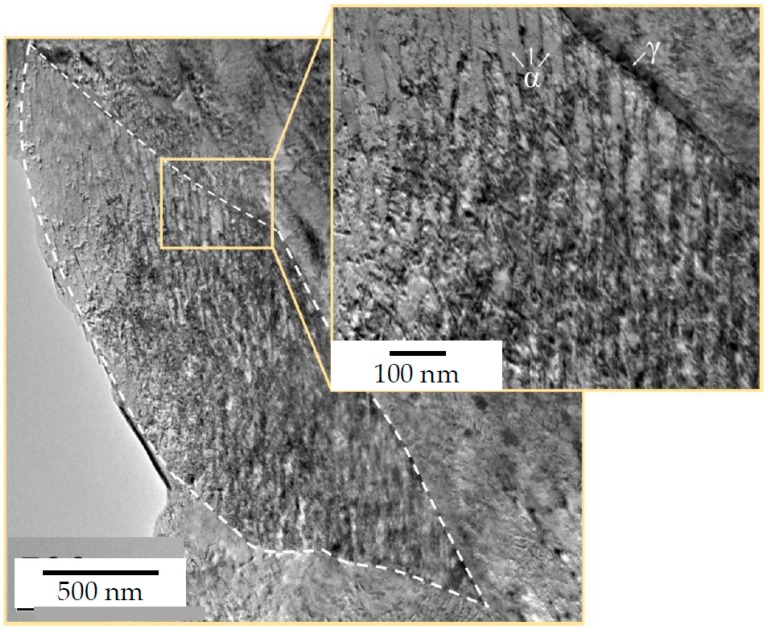
HRTEM Micrograph of the steel after intercritical annealing/heat treatment route ②, showing a single sheave of bainite, with a typical lenticular morphology; when observed in bright field mode, the bainitic ferrite plates (α) appear light, and the retained austenite (γ) dark.

The ageing process can be well monitored by measuring the retained austenite content after different heat treatments, the results of which are summarised in Table 3:

The steels’ microstructure after heat treatment ① was observed using light and field emission scanning electron Microscopy (FESEM), as can be seen in Figure 2 and Figure 3 respectively. Both methods of characterisation show a significant fraction of retained austenite present in comparably coarse, as well as micron-sized blocks. The internal structure and interphase of the austenitic/bainitic regions is shown in the HRTEM micrograph in Figure 4. The result is similar to the HRTEM micrograph of microstructure after ②, which is shown in Figure 5, as these heat treatments result in a comparable amount of retained austenite.

**Figure 6:**
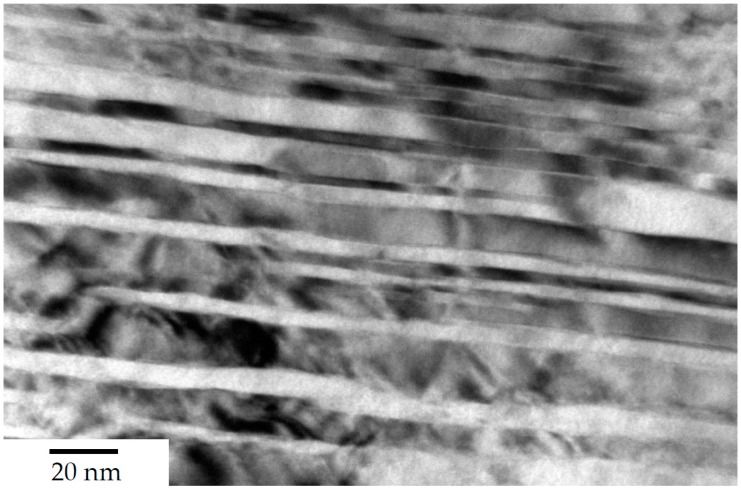
HRTEM image of fine bainitic microstructure of steel A0, heat treated according to route ③. Showing individual bainitic ferrite plates and some particles of retained austenite. When observed in bright field mode the bainitic ferrite plates (α) appear light, and the retained austenite (γ) dark.

The retained austenite initially present has later been shown to decompose, resulting in some remarkably fine structures, as can be seen in Figure 6. The main benefit is that it does provide an appreciable increase in toughness, and a much less pronounced notch sensitivity when compared to either of the other two heat treatments. It should be noted at this point that intercritical annealing is not necessary when heat treating from an initially annealed microstructure. A precise determination of the characteristic length is difficult, as the structure is highly hierarchical due to the continuous cooling heat treatment, which introduces subunits with a thickness of as much as 50 nm or more, and as thin as a few nanometers. The very fine structure comprises about 25% of all bainitic ferrite plates, the majority of which were formed between 180 °C and 80 °C, with an average thickness of about 25 nm.

### 3.2. Toughness

Within the vast majority of commercial steel grades there is at least some correlation between the fracture toughness and impact properties, such that attempts were made to predict one property based on the other, like, for instance, British Standard BS PD6539:1994.

In the case of low temperature carbide free bainitic microstructures, we observe a very low impact toughness, despite the often high values of fracture toughness [27]. Analogous to the tensile properties, the toughness also depends vastly on the retained austenite. During a fracture test, the specimen is subjected to a sharp crack and a small amount of retained austenite is transformed into martensite, and the resulting volume expansion acts favourably by closing the crack tip. This effect has long been known, even from the original studies on metastable austenitic steels. 

**Figure 7:**
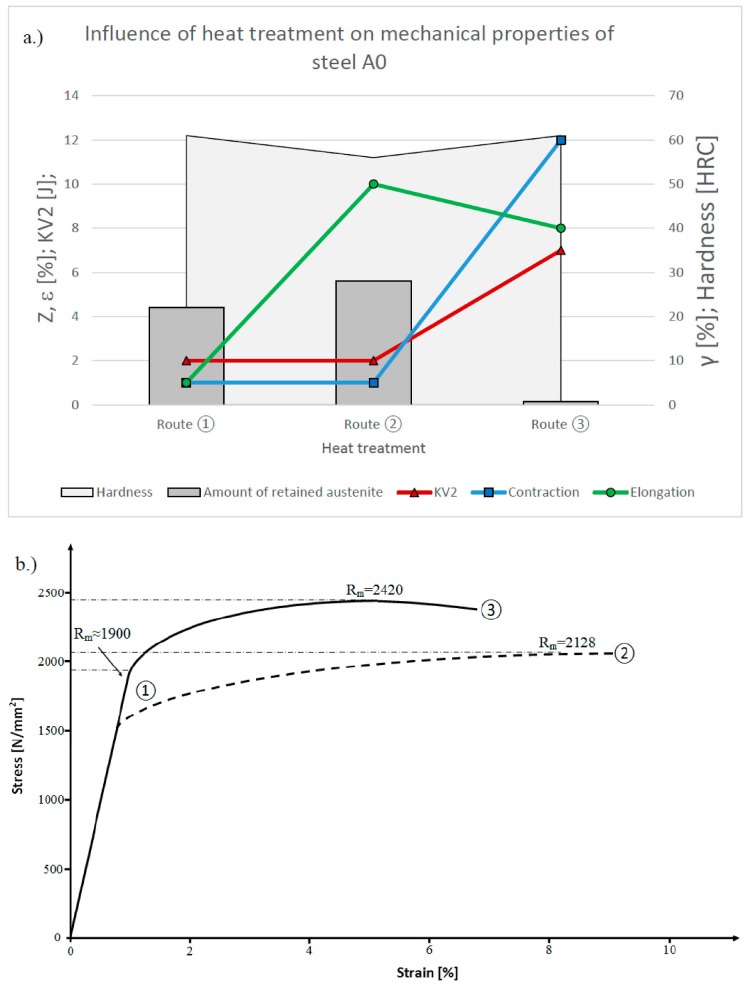
Balance of mechanical properties, of steel A0 in relation to heat treatment: (**a**) general overwiev, (**b**) corresponding tensile curves.

**Figure 8:**
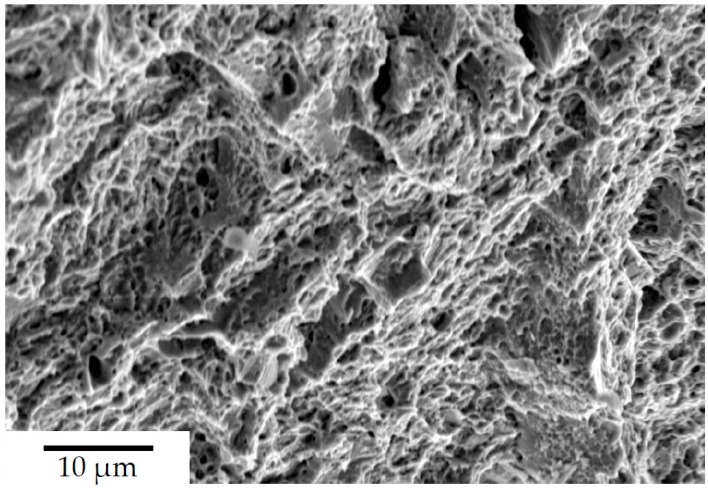
Fracture surface of impact specimen made from steel A0, after heat treatment ③.

During an impact test, however, we have a comparably wide notch; the notch tip acts as a stress concentration, so that the retained austenite in the comparably larger adjacent region transforms into martensite. This can occur even under the influence of elastic strains, therefore, untempered martensite with a very high carbon content is formed during such tests in almost all low temperature bainitic steel grades. This low impact toughness of less than 10 J is so common it is even considered an intrinsic property of low temperature carbide free bainitic microstructures [28]. There are, however, a variety of methods by means of which the formation of brittle untempered martensite could be avoided, whereby the most straightforward is by tailoring the chemistry to obtain a low Retained Austenite (RA) volume fraction after quenching to bainite, and low temperature tempering, as shown in Figure 7. Such behaviour is consistent with other results obtained on similar steels [29]:

Heat treatment ③ applied to steel A0 reduces the volume fraction of retained austenite far below critical levels, and, thereby, increases the toughness markedly to about KV2 7J at the same hardness, and with almost no brittle character at the fracture surface, as can be seen in Figure 8:

## 4. Discussion

Heat treatment ① makes the best use of the KAB principle, and produces a high hardness and quite low ductility, as well as a very pronounced notch sensitivity, whereas ② enables the steel to retain a larger portion of austenite, and thereby utilise a more pronounced transformation induced plasticity-TRIP effect. The steel exhibits a higher elongation, which is almost entirely uniform with a negligible contraction. Fracture of carbide free bainitic steels is, therefore, thought to occur in a brittle manner when this threshold of about 10% is reached [30,31]. Therefore a volume fraction of 10% can be considered as the critical value, where the steel is neither tough nor ductile, as shown previously in [29]. Heat treatment ③ demonstrates the limits of temperatures at which this steel can be transformed and the fine scale of bainitic ferrite plates which can be obtained thereby. The resultant microstructure after route ③ combines a very high hardness with almost no loss in ductility, whereby a significant amount of contraction is observable during tensile testing. Unfortunately, natural ageing doesn’t provide any benefits in terms of an additional increase in hardness for steel A0. The required time of 12 days in order for the bainite reaction to complete in steel A0 is thought to be a consequence of the slow growth of bainitic ferrite at room temperature. The growth rate is known to be significantly slower compared to martensite [32], and some studies suggest the diffusion of carbon occurs during growth of bainitic ferrite plates [33,34,35]. The latter seems unlikely when bulk diffusion is considered, which supports strongly the view that the bainite forms via a displacive mechanism.

The formation of bainite at room temperature occurred within the prior retained austenite blocks and thicker films, therefore, some residual micro stresses are likely present. The latter are known to promote the selection of favourably oriented crystallographic variants [36,37]. This could explain the change in sheave morphology to comparably straight sheaves, comprised of subunits with identical orientation.

Low impact toughness could be considered as the main drawback of hard carbide free bainitic steels, and, thus, its biggest inhibitor to gaining widespread industrial implementation [38]. As mentioned before, this is an inherent trait of mixed microstructures containing brittle martensite [39]. Heat treatment route ③ offers a possibility to mitigate this issue. When plotting the mechanical properties in relation to the retained austenite content, as can be seen from data in Figure 7, this phenomenon is confirmed, as s high amount of sufficiently stable retained austenite obtained via route ② favours homogeneous elongation, whereas the heat treatment acc. to route ③, with a low retained austenite content, exhibits a significant increase in contraction and impact energy. In this sense, additions of Cr are especially crucial, as this element increases the amount of retained austenite and is known to be detrimental to the toughness of low carbon bainitic steels [40].

## 5. Conclusions

It can be seen that, through the development of a very fine carbide free bainitic microstructure, very high strength levels can be obtained within steels of lean composition. The heat treatment times are, for now, very long, but only present a marginal increase in cost as it is performed at room temperature. It is assumed that the required times for natural ageing can be shortened significantly without a decrease in hardness, by using alloys with lower contents of austenite stabilising alloying elements. The strength of the current steel originates mainly from the very fine structure and dislocation strengthening mechanisms, whereby the role of carbon needs to be elucidated further. It is, however, reasonable to assume a further increase in strength by increasing the volume fraction of the room temperature bainite.

Additionally, when the retained austenite volume fraction is reduced to a very low level, the steel can retain high levels of toughness, despite a high hardness and the use of low tempering temperatures. The observed toughness is thought to be correlated to the theoretically immense dislocation density, whereby the majority are introduced by the formation of bainite subunits, and, therefore, considered to be significantly more mobile when compared to those introduced via severe plastic deformation, thereby further outlining that phase transformations offer several advantages, and, therefore, present the preferred route for obtaining nanostructures with the possibility of large scale production, although the scaling of the newly developed steel grade to industrial charges is currently the main goal.

Further research work should also be directed toward the characterisation of complex mechanical properties such as fracture toughness, wear resistance, and high strain rate behaviour of the newly developed steel.

## 6. Patents

Part of the work reported herein has been filed for a patent at the Slovenian Patent Office under nr. P-201900181.

## Figures and Tables

**Table 1:** Strengthening mechanisms in different steels, partially adapted from [8].

Steel/Production Route	Ref.	Characteristic Length (nm)	$\mathrm{Fraction}\;\mathrm{of}\;\mathrm{Internal}\;\mathrm{Boundaries}\;S_{V}\;\left(nm^{-1}\right)$	σ (MPa)
Nanostructured carbide free lower bainite—Superbainite	[9]	t=20−40	$\frac{2}{t}=0.05\;to\;0.1$	>2100
Nanostructured kinetically activated bainite (steel A0-current work)		t=5−50	$\frac{2}{t}=0.04\;to\;0.2$	>2600
Formation of fine bainite	[10]	$\bar{L}=200-400$	$\frac{2}{\bar{L}}=0.005\;to\;0.01$	<960
Nanostructured martensite	[11]	$\bar{L}=100-200$	$\frac{2}{\bar{L}}=0.01\;to\;0.02$	1530
Nanostructured martensite/austenite dual phase steels	[12]	$\bar{L}=25$	$\frac{2}{\bar{L}}=0.08$	2000 *
Reverse transformed austenite	[13]	$\bar{L}=200-600$	$\frac{2}{\bar{L}}=0.005-0.0016$	1400
Mechanical milling	[14]	$\bar{L}=20$	$\frac{2}{\bar{L}}=0.1$	2850
Nanoparticle strengthening	[15]	$N_{V}=\frac{1.1\times 10^{24}}{m^{3}},r=1.25\;\mathrm{nm}$	$4\pi r^{2}N_{V}=0.011$	800
ARB accumulative roll bonding	[16]	$\bar{L}=420$	$\frac{2}{\bar{L}}=0.005$	870
HPT high pressure torsion	[17]	$\bar{L}=100$	$\frac{2}{\bar{L}}=0.02$	1570
ECAP Equal channel angular processing	[18]	$\bar{L}=200$	$\frac{2}{\bar{L}}=0.01$	1150

* Measured after partitioning treatment, also contains carbides.

**Table 2:** Nominal compositions of characterised steels (in w %).

	C	Si	Mn	Mo	Cr	V	Al	Ti	Nb	Other	Fe
Steel A0	0.7	1.2	2.5	0.6	1.8	0.22	1.5	0.015	0.02	<1	Bal.

**Table 3:** Phase fraction of High C-Al KAB steel, “Steel A0” after different heat treatments.

Sample	Phases (vol.%)	Hardness (HRC)
Continuously cooled ①	Gama = 22%, Alfa = 78%	61–62
Intercritically annealed ②	Gama = 27.8%, Alfa = 72.2%	56–57
Aged Naturally for 12 days ③	Gama = 0.7%, Alfa = 99.3%	61–62
